# Principles of Management in Isolated Dorsal Distal Interphalangeal Joint Dislocations

**Published:** 2014-09-19

**Authors:** Stella Chung, Aditya Sood, Edward Lee

**Affiliations:** Division of Plastic and Reconstructive Surgery, Department of Surgery, New Jersey Medical School, Rutgers University, Newark, NJ

**Keywords:** DIP dislocation, finger dislocation, dorsal dislocation, closed reduction, finger trauma

**Figure:**
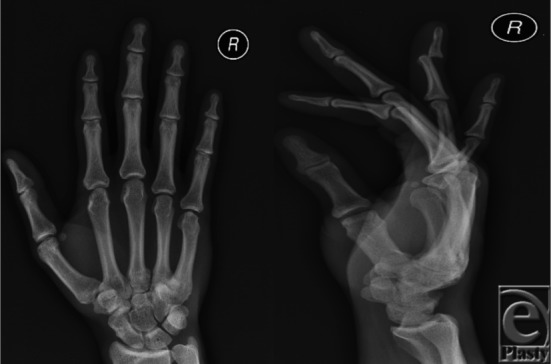


## DESCRIPTION

A 26-year-old right-handed man presented with pain in his right ring finger after a physical altercation. Deformity and swelling in the distal interphalangeal (DIP) joint was evident. There was no open injury or sensory loss; flexion and extension were limited secondary to pain. X-ray showed posterior dislocation of the ring finger DIP joint without fracture.

## QUESTIONS

1. **How common are isolated DIP joint dislocations and what is the usual mechanism of injury for this particular injury?**
2. **What are the important physical examination findings when assessing DIP joint dislocation?**
3. **How do you reduce a dorsal DIP joint dislocation?**
4. **What is the surgical and applied anatomy of the DIP joint?**

## DISCUSSION

Dislocations of the DIP joint of the fingers are often associated with fracture, tendon rupture, and/or proximal interphalangeal (PIP) joint involvement. Closed isolated DIP joint dislocations are exceedingly rare. According to a literature review by Abouzahr and Poblete,^1^ 12 cases of DIP joint dislocation have been published between 1940 and 1997. Patients often acquire this pattern of injury from playing aggressive ball-catching sports such as volleyball or basketball. Upon axial loading and hyperextension of the fingertip, the volar plate of the DIP joint tears and the joint may be displaced. The direction of dislocation is more commonly dorsal; however, lateral or volar types may also be encountered.

When examining a DIP joint dislocation, it is important to assess for any associated soft tissue injury, loss of sensation, active/passive motion, capillary refill, and injuries to other digits and joints of the extremity. Furthermore, examination of terminal extensor tendon and flexor digitorum profundus (FDP) injury and fracture dislocation must be included. Dorsal dislocations of the DIP joint are often open; therefore, evaluation for any open injury is warranted. Open injuries commonly present with a transverse laceration in the flexion crease, and this must be treated as a contaminated joint. Radiographic evaluation should include anteroposterior and lateral views of the injured finger to evaluate for fracture or other joint derangements.

The first line of treatment for a dorsal DIP dislocation without fracture or tendon injury is an attempted closed reduction. With local anesthesia, axial traction is applied and the distal phalanx is brought into hyperextension, while pushing the base of the distal phalanx volarly back into its natural position. It is then immobilized in slight flexion with a dorsal splint for 2 to 3 weeks. Closed reduction and splinting prevents redislocation during early collateral ligament healing.^2^ Other treatment options for DIP dislocation include closed reduction with percutaneous pinning or open treatment of the dislocation. Open reduction and fixation is indicated in chronic injury (>3 weeks) and irreducible dislocations.^3^ Stiffness and/or pin tract infection may be encountered with the mentioned treatment options.

The DIP joint is a bicondylar joint stabilized on each side by proper and accessory collateral ligaments and volarly by the volar plate. The ligaments provide static stability of the joint. The balanced forces of the terminal extensor tendon and the long flexor tendon provide dynamic stability. The DIP joint lacks checkrein ligaments and its volar plate is weakly confluent with the distal extent of the flexor digitorum superficialis tendon. These factors may contribute to volar plate detachment with dorsal dislocations.^4^ Irreducible DIP joint dislocations are generally caused by FDP tendon dislocation or interposition of the volar plate into the DIP joint.^5^ Types of FDP tendon dislocation as summarized by Ghobadi and Anapolle^6^ include the following: entrapment of the FDP tendon behind a condyle of the middle phalanx, volar plate avulsion from the middle phalynx with interposition into the joint, a buttonhole tear through the volar plate, and entrapment of the distal middle phalynx into a longitudinal split of the FDP tendon. The treatment for irreducible dislocations requires an open surgical technique

DIP joint dislocations are often underappreciated and may present late.^3^ Isolated DIP joint dislocations without tendon or PIP joint involvement are rare. Our patient presented with an isolated, closed, right ring finger DIP joint dislocation soon after a physical altercation. After a closed reduction treatment, he displayed intact FDP function and DIP joint extension and was appropriately immobilized in slight flexion of the joint.
